# Perceptions on the effect of small electric fans on comfort inside bed nets in southern Ghana: a qualitative study

**DOI:** 10.1186/s12936-016-1614-x

**Published:** 2016-12-01

**Authors:** Mulako S. Jaeger, Olivier J. T. Briët, Joseph Keating, Collins K. Ahorlu, Joshua O. Yukich, Samuel Oppong, Peter Nardini, Constanze Pfeiffer

**Affiliations:** 1 Swiss Tropical and Public Health Institute, Socinstr. 57, 4002 Basel, Switzerland; 2 University of Basel, Petersplatz 1, 4003 Basel, Switzerland; 3 Center for Applied Malaria Research and Evaluation, Tulane University School of Public Health and Tropical Medicine, 1440 Canal St, Suite 2301, New Orleans, LA 70112 USA; 4 Noguchi Memorial Institute for Medical Research, College of Health Sciences, University of Ghana, P.O. Box LG581, Legon, Ghana; 5 National Malaria Control Programme, P.O. Box KB 493, Korle-bu, Accra, Ghana; 6 Green World Health Net, 307 Amherst Dr. SE, Albuquerque, NM 87106 USA

**Keywords:** Malaria, LLIN, LED, ITN, Fan, Solar power, Ghana, Key informant interviews

## Abstract

**Background:**

Long-lasting insecticidal nets (LLINs) are known to be highly effective in reducing malaria transmission, morbidity and mortality. However, among those owning an LLIN, use rates are often suboptimal. A reported barrier to bed net use is discomfort due to heat. This qualitative study was part of a larger evaluation conducted in communities without electricity in rural Ghana to assess whether 0.8 W solar powered net fans can increase net use.

**Methods:**

Twenty-three key informant interviews with household heads in the study communities in Shai-Osudoku District, southern Ghana, were conducted from July to August 2015. The purpose of the interviews was to obtain insight into perceptions of participants about the net fan system in relation to LLIN use.

**Results:**

While all study participants reported using LLINs, with mosquito nuisance prevention as the prime motivation, heat was also mentioned as a key barrier to net use. Respondents appreciated the net fans because they improved comfort inside bed nets. The LED light on the fan stand became the main source of light at night and positively influenced the perception of the intervention as a whole.

**Conclusion:**

The general acceptance of the net fan system by the study participants highlights the potential of the intervention to improve comfort inside mosquito nets. This, therefore, has a potential to increase bed net use in areas with low access to electricity.

## Background

The World Health Organization (WHO) World Malaria Report 2015 indicates that about half of the world´s population (3.4 billion people) live in areas at high risk of malaria transmission [1]. Malaria transmission occurs in all regions of Ghana, putting the entire population at some risk of transmission, and malaria is the leading cause of death among children under the age of five and accounts for about 38.1% of all outpatient hospital visits [2].

One of the key malaria control interventions emphasized in the Roll Back Malaria (RBM) initiative is the use of long-lasting insecticide-treated nets (LLINs) to prevent infection [3]. LLINs have shown to reduce the burden of malaria in Ghana [4]. Ghana implemented these protective measures through its National Malaria Control Programme (NMCP). Enormous efforts to educate the local communities on the causes of malaria and subsequently increase use of bed nets were made after the launch of the RBM initiative [2]. In 2001, a national policy to promote the use of LLINs was adopted in Ghana. Mass campaigns, child-health week celebrations, voucher schemes targeting pregnant women, routine distribution of nets through child welfare clinics and antenatal clinics were all used to increase LLIN awareness and use. These strategies focused on making LLINs affordable and available through the commercial sector and subsidized prices [5]. Subsidized LLINs were also made available through free campaigns during national immunization days and through non-governmental organizations [5]. While the vulnerable groups were being targeted, the rest of the population, including older children and adults who also contributed to malaria transmission, were not [6]. In 2010, Ghana adopted a universal coverage policy. In 2011, the Volta and Eastern region were chosen as pilot regions for a mass campaign targeting the entire population. A registration exercise was done for all households; names, ages, and numbers of sleeping spaces of every member of the household were recorded. LLINs were allocated based on a ratio of two persons to one net.

Between May 2010 and October 2012, approximately 12.5 million LLINs were distributed in all 10 regions of Ghana through a national universal mass distribution hang-up campaign [7]. Despite universal LLIN coverage [8] only 60% of bed nets within the households that had at least one LLIN were being utilized [7]. In addition, net use rates dropped to nearly 50% for children under the age of five and pregnant women [7].

One of the reported reasons for the low usage in bed nets is heat [9]. The thermal discomfort experienced at nighttime depends on several factors such as humidity, temperature and air velocity inside the net as well as the house [9]. While mosquito net manufacturers try to increase the ventilation by making the mesh size as large as possible, they are limited by the small size of mosquitoes. In the light of the above, low power fans were suggested to increase comfort inside nets [10–13]. Considering that, especially in rural areas in sub-Saharan Africa (SSA), more than 85% of the population does not have access to the grid, solar cells may provide the electricity source for small solar powered fans [10, 13]. The cost of solar energy has decreased over the past decades and costs less than 2 US$ per watt [14].

Thus, a solar powered fan system with the fan on a pedestal that allows the net to be tucked in with the fan inside the net, named the ‘Bͻkͻͻ net fan system’ (Bͻkͻͻ in the local language Twi has a double meaning: “I am well” (healthy) and “I am cool” (literally). The term was selected building on discussions with local partners in Accra), was developed [10, 13]. To better understand whether the Bͻkͻͻ net fans can increase bed net usage, a randomized cross-over trial was implemented in rural communities without electricity in southern Ghana. A qualitative study was conducted to provide additional information about individuals’ perceptions related to the Bͻkͻͻ net fan system, and whether these perceptions influence net use. In this paper, the findings from the qualitative study are presented; the quantitative findings are reported elsewhere [15].

## Methods

### Study design

The cross over trial, in which the qualitative study was nested, a census at the project initiation meeting with the communities of two villages in Shai-Osudoku District in southern Ghana generated a sampling frame of 104 households. The inclusion criteria included households with at least two members in residence. In a next step, 83 households were randomly selected into three groups. Each group consisted of 26–30 households. It was observed that almost all households still had bed nets from the mass distribution campaign done in 2013. However, most of these nets were in poor condition. A screening survey was conducted and new LLINs were distributed to all participating households. Group 1 received the Bͻkͻͻ net fan system, one 0.8 W fan fitted with a 0.1 W LED light for each household member, Group 2 received a water filter as an alternate intervention to serve as a comparison, and Group 3 served as control for intense study contact. After four months, Groups 1 and 2 crossed over interventions. Throughout the study, households in Groups 1 and 2 were visited fortnightly, and the head of the household or an adult was subjected to a questionnaire with close-ended questions about the use of mosquito nets and barriers and facilitators to use of mosquito nets. During the month before cross-over, key-informant interviews with household heads in Groups 1 and 2 were conducted to provide a more detailed insight into the local perceptions and contextual factors and their influence on the use of the solar powered electric fans as well as bed nets. The questions in the interviews centered around three main topics: (1) use of bed nets, (2) perceptions regarding fans and (3) perceived benefits of and barriers to fan use.

### Study site

The study was conducted in the villages of Apese (Abuminya) and Amanfrom in Shai-Osudoku District. The communities were selected due to their limited access to electricity. Shai-Osudoku District is located in the southeastern part of Ghana in the Greater Accra Region. The Greater Accra Region was identified as one of the regions with a low use of ITNs [16]. Shai-Osudoku District covers an area of about 721 square kilometers and has a population of approximately 52,000, based on the latest census figures from the Ghana Statistical Service [17]. It is a predominantly rural area with 76.4% of the population living in rural areas [17]. The vegetation is mostly coastal savannah shrubs with few thickets. The region falls within the dry coastal equatorial climatic zone with temperatures ranging between 20° and 30° Celsius and annual rainfall ranging from 635 mm along the coast to 1140 mm in the northern parts. There are two notable rainfall peaks, one in June and one in October. The rainfall season between April and July is associated with the major farming activities in the region [18]. In terms of religion, the region is dominated by Christians (83.0%), followed by Moslems (10.2%), people with no religious affiliation (4.6%) and 1.4% traditional religion advocates [19]. The major ethnic groups in the region are the Akan (39.8%), Ga-Adangme (29.7%) and Ewe (18%) [16]. In the selected study villages, the Ewe were predominant.

### Participant selection

Participants for the key informant interviews were purposively selected based on whether the respondent spoke the local language Twi. This was because the local research assistant spoke Twi, but not Ewe. Interviews were conducted with the household heads or, in their absence, a member of the household who was at least 18 years of age. Using the rule of saturation [20], 13 participants from Group 1 and 10 from Group 2 were interviewed. There were more respondents in Group 1 than in Group 2 in order to reach data saturation. No participants were selected from Group 3.

### Data collection

The key informant interviews were carried out between July 2015 and August 2015. Interview guides were pre-tested and revised. Data were collected by MJ (female with Zambian nationality) who also took additional notes during the interviews. The research assistant (male with Ghanaian nationality) translated the interview questions and probes between Twi and English directly to ensure correct meaning. A member of the community accompanied the data collection team.

### Data analysis

Data from the interviews were translated from the local language Twi into English by the research assistant and recorded onto an audio device. The audio files were transcribed verbatim by MJ directly after each interview with the help of the research assistant. Data were reviewed and any ambiguous interpretations were clarified during and after the transcribing. Majority of the study participants made some additional comments after the tape recorder was turned off; these conversations were also documented. Data were analyzed using framework analysis [21]. Codes and themes were managed using MAXQDA 12.0 data analysis software. A codebook was developed based on the research questions as well as on themes emerging from the transcripts. Original transcripts and field notes were reviewed when more contextual information and clarity was needed during coding and analysis.

## Results

A total of 23 respondents representing households were interviewed (Seventeen were household heads and the remaining were either spouses or sons and daughters over the age of 18): 48% were female and 52% were male (Table 1). Detailed socio-demographic characteristics are presented in Table 1.

**Table 1:** Socio-demographic characteristics of respondents in selected households in villages of Apese and its settlements and Amanfrom, Shai-Osuduko district, Greater Accra, Ghana, 2015

	Group 1 intervention (N)	Group 2 control (N)
*Age*		
<30	4	1
30–39	1	4
40–49	2	3
50–59	2	0
60–69	2	2
70+	2	0
*Gender*		
Male	7	5
Female	6	5
*Ethnicity*		
Ga Adangbe	1	1
Ewe	11	7
Other	1	2
*Marital status*		
Single	1	0
Married/cohabiting	10	10
Widowed	2	0
*Religion*		
Traditional	1	0
Christian	11	9
Islamic	1	0
None/other	0	1
*Highest level of education*		
Attended	4	3
No formal education	1	1
Primary school	2	1
Middle JHS	62	5
Secondary/tertiary	6	
*Profession*		
Unemployed	1	0
Farming	10	8
Trading	0	2
Other	2	0
Total	13	10

### Use of bed nets

To provide a general picture of the community’s attitude and practice related to bed net usage, participants were questioned about their sleeping behaviour. In general, bed net usage was very high. All of the respondents and their families reported to have slept under the bed net the night before the interview. Mosquito nuisance was the predominant reason stated for the high bed net use among the participants.

“I slept inside the mosquito net because of mosquitoes. If you don’t sleep inside the net, mosquitoes will bite you. So to prevent this I sleep inside the net” (Male, 51 years old, Group 1)

“My wife and children. We all slept under the bed net because of mosquitoes” (Male, 32 years old, Group 2).

Few respondents (3/23) reported that mosquitoes are not only a biting nuisance but their noise makes it almost impossible for them to sleep the entire night and this is why most of the community members preferred to sleep under bed nets.

The majority of the respondents (18/23) were worried about getting malaria and believed that it was more important and beneficial to sleep under a mosquito net because sleeping under a mosquito net is a good way to protect themselves from malaria.

“If I sleep under the bed net always, it will prevent me from getting malaria” (Male, 32 years old, Group 1).

“That is what you are doing by providing mosquito nets. Sleeping inside mosquito nets prevents malaria” (Male, 72 years old, Group 1).

However, many of the respondents (15/23) cited that seasonality, linked to temperature and mosquito density determined net use and outdoor sleeping behaviour.

“When there is heat, there are not so many mosquitoes so we normally sleep outside” (Male, 32 years old, Group 2).

“Nothing prevents me from using the nets unless maybe there are not many mosquitoes around. Also when the wind is blowing I can sleep outside without the net” (Male, 44 years old, Group 1).

Some of the respondents (8/23) stated that outdoor sleeping is common throughout the hot and dry season, which runs from October through to April in the study area. The respondents mentioned heat as the predominant reason for sleeping outdoors, explaining that the wind blows during the night in the hot and dry season making it more comfortable to sleep outdoors as opposed to sleeping indoors.

When asked the reasons that would prevent a member of the community from sleeping under a bed net, nearly all of the respondents (22/23) stated discomfort essentially due to heat as the main reason for non-bed net usage.

“A lot of people complain about the heat in the net” (Male, 32 years old, Group 1).

“When there is heat I don’t sleep in the net. I fold the net upwards and sleep” (Male, 44 years old, Group 1).

“I sleep inside the nets more often but sometimes when it’s hot I do not sleep inside” (Female, 32 years old, Group 2).

On the other hand, when asked “What motivates you to sleep under a bed net?”, a minority of the respondents (6/23) mentioned that the cold motivates them to sleep under a bed net.

“When the weather is cold” (Female, 32 years old, Group 2)

“Because of coldness too” (Male, 42 years old, Group 1)

“Because of coldness and mosquitoes” (Female, 32 years old, Group 2)

### Use of fans

The majority of the participants in Group 1 reported to have used the fan the night before the interview. The respondents mainly noted heat as the reason for using the fans. Two thirds of the participants (16/23) also cited the perceived benefit of driving the mosquitoes away as a reason for using fans.

“There was heat in the room that is why I used the fan” (Male, 44 years old, Group 1)

“Because of heat and mosquitoes” (Female, 18 years old, Group 1).

“Sometimes the fan also drives the mosquitoes away” (Female, 22 years old, Group 1).

However, most of them (9/13) stated that they used the fan outside the bed net explaining that the air produced from the fan was enough to reach the bed net.

“Our room is not that big and such the distance between the fan and the bed is small. It is not really necessary to place the fan inside the net since the air from the fan reaches us under the net” (Male, 51 years old, Group 1).

Another respondent noted that the rest of the family also used the fans outside the net.

“They fix the fan outside the net but it blows on them in the net” (Female, 65 years old, Group 1).

### Perceived barriers to fan use

A few respondents (3/13) noted that they did not use the fans the night before the survey due to the cold weather. The interviews were conducted at the end of the rainy season when temperatures are cooler. The same was mentioned in informal discussions with the respondents. Because of the cooler season, placing the fan under the bed net was sometimes linked to experiences of chills and chest pains. However, respondents stated that they would place the fans under the bed nets during the hot season.

“Yes. If there is heat, we always use it but now that there is cold, we don’t always use it. We only use the fan during the hot season” (Male, 53 years old, Group 1).

“I don’t really like it when the weather is cold. I have placed the fan outside the net so when it is cold, I don’t use the fan” (Male, 51 years old, Group 1).

All respondents from Group 1 reported using the LED lights on the fan stand regularly. Among them, three stated that due to low temperatures they utilize the LED light on the fan stand only without using its cooling function.

“No, because the weather is cold now but I used the light” (Male, 53 years old, Group 1).

### Perceived benefits of fan use

Despite the reported high LLIN use rate in both Group 1 and 2, the study participants acknowledged the importance of the small powered electric fans. Two thirds of the respondents from Group 2 (6/10) stated that the fan would make them sleep under a bed net in the hot season.

“I’m praying that I get a fan under the net. That would help me sleep in the net even when it is hot” (Male, 40 years old, Group 2).

The respondents from Group 1 explained that the fans improved comfort and therefore increase net usage in the hot season and reduce outdoor sleeping.

“Yes. I use it [the fan]. For instance before the introduction of the fan we used to sleep outside till about 10:00 pm and when the weather becomes cool, then we go inside to sleep” (Male, 53 years old, Group 1).

“If you don’t sleep in it, the weather is hot, and you cannot sleep outside. If you sleep outside the mosquitoes will bite you. But if you sleep under the fan and the net is also in the room, you will feel very comfortable” (Male, 74 years old, Group 1).

“The fan enhances frequent use of the nets” (Male, 42 years old, Group 1).

While the majority of the respondents in Group 1 (12/13) noted that heat was the main motivation for using the fans, many of them (8/13) also stated that the LED light on the fan stand was an important motivator for using the fans.

“Now I sleep inside the net with the fan and light so it’s better than when I didn’t have the fan” (Male, 44 years old, Group 1).

The LED light on the fan stand served as an important feature for the study participants. The majority of respondents in Group 1 (10/13) also expressed that the light on the fan stand had become their only source of light. All of the respondents in Group 1 (13/13) reported that they used the LED light the night before the interviews. Approximately two thirds of the respondents indicated that they used the light the entire night, for example for studying or feeding babies.

“When I switch it on, it stays on till the following morning” (Female, 60 years old, Group 1).

“I use the light always. I even use it to sleep” (Female, 28 years old, Group 1).

When asked about potential improvement of the fan system, more than half of the respondents in Group 1 (7/13) suggested increasing in size of the fan as well as the brightness of the light.

“If the fan could be made bigger I think that would be better because this one is too small” (Male, 51 year old, Group 1).

“The light should also be made bigger so that it can brighten the entire room” (Female, 65 year old, Group 1).

“Oh, if the size of the bulb is improved we will appreciate it but even with this we are still managing it” (Male, 53 year old, Group 1).

When asked what could be done to increase or improve bed net use, about half of all the respondents (12/23) cited that the provision of fans would increase bed net use rate. No viewpoints opposing the fans were mentioned by the respondents.

“Oh if some people are not using the nets, then it may be due to the fact that they did not get access to fans, so they are not sleeping in the net because of heat, But those of us who have the solar panel, we sleep inside the net” (Male, 53 year old, Group1).

“If the fan is part of the net it is good” (Female, 65 year old, Group 1).

“By providing them with fans” (Female, 32 year old, Group 2).

## Discussion

The findings of this study provided insights into bed net as well as fan use by community members in Shai-Osudoku District, southern Ghana. In contrast with the 2014 Ghana Demographic and Health Survey (DHS), which indicated that mosquito net use in the late rainy season (September to November) was low in the Greater Accra region [22], mosquito net use by study participants was high. The Ewe, a minority in the Greater Accra region, has a reputation of common bed net use going back generations. Nevertheless, a variety of barriers to bed net use exists. The findings in this study support earlier research highlighting discomfort due to heat and (low) perceived mosquito density as barriers to bed net use [9, 23–25]. In addition, this study also indicated that outdoor sleeping is common throughout the hot and dry season, which runs from approximately October to April in the study area. Similarly, a study conducted in the northern part of Ghana found that outdoor sleeping and other night-time activities were extensive during the hot and dry season [26].

The findings of this qualitative research revealed that the fans were well received and utilized in the group that received the fans. In addition, the fan reportedly improved comfort inside the bed nets. This is in support of the hypothesis that providing air circulation within mosquito nets through the installation of fans inside the nets improves comfort. Furthermore, most respondents indicated that the LED light on the stand had become their main source of light. Through observations from the research team it was evident that the LED light was an incentive to use the fan. The findings confirmed that the study participants in Group 1 appreciated the fans and encouraged further distribution.

The study participants in Group 2 often reported to have heard from their neighbors how the fans had positively changed their lives. When they were asked to be more specific, they stated that the LED lights on the fan stands had enabled their neighbors to perform several tasks within their households at night. They also mentioned that they had heard that the fan helped to drive the mosquitoes away and cool off their rooms.

In these villages without connection to the electricity grid, the LED lights on the fan stands were highly valued, as they became study participants’ main source of light inside the room. This finding was supported by observations from the study team who offered to sell a limited number of 39 household light sets (12 V bulbs, switch box and 12 V socket) at a price of 28 Ghanaian Cedis (US$ 7.3) which was the original price from the solar panel supplier —all sets were sold within two days.

### Study limitations

Participants were selected based on the ability to speak Twi since it was not possible to find an interpreter who spoke both Twi and Ewe languages at the time of the interviews. Key informant interviews were conducted with participants who had been exposed earlier by the same project to a series of survey questions about barriers and facilitators to mosquito net use. This might have biased their answers. When asked “Where did you learn about the importance of using nets?”, over half of the respondents (14/23) cited the study trial as a source of information for using bed nets. In addition, the presence of a field assistant recruited from the local community may have biased respondents into giving socially desirable answers.

## Conclusion

Bed net use was common in this community. The interviews gave a mixed picture about the suitability of the design of the fan system with a (low) power rating of 0.8 W. While half of the participants desired a larger sized fan, more than half of the respondents reported that they placed the fan outside the bed net because the air produced from the fan was enough to reach the bed net.

The LED light was an important added feature for the communities that were mainly relying on battery powered torches and kerosene lamps as sources of light at night prior to intervention. Thus, LED lights may have contributed importantly to the acceptance of the Bͻkͻͻ bed net fan system and use behaviour.

Respondents’ strong acceptance of the fan suggests the potential of the intervention to increase net use in other study areas that are characterized by hot weather and low bed net utilization rates. Future studies should evaluate the interaction between weather (hot vs. cold periods) and fan exposure on net use.

## Abbreviations

DHS: Demographic and Health Survey
IRB: institutional review board
LED: light emitting diode
LLIN: long-lasting insecticidal net
NMCP: National Malaria Control Program
RBM: Roll Back Malaria
SSA: sub-Saharan Africa
US: United States
WHO: World Health Organization
